# Does clinical examination aid in the diagnosis of urinary tract infections in women? A systematic review and meta-analysis

**DOI:** 10.1186/1471-2296-12-111

**Published:** 2011-10-10

**Authors:** David Medina-Bombardó, Antoni Jover-Palmer

**Affiliations:** 1 Manacor Health Center. Majorca Primary Care Department. Balearic Institute of Health Manacor, Simó Tort s/n, 07500 Manacor, Balearic Islands, Spain; 2 Arquitecte Bennasar Health Center, Majorca Primary Care Department. Balearic Institute of Health, Avda Gaspar Bennàzar 9, 07004 Palma, Balearic Islands, Spain; 3 Unit of Reseach, Majorca Primary Care Department. Balearic Institute of Health, Reina Esclaramunda 9, 07003 Palma, Balearic Islands, Spain

## Abstract

**Background:**

Clinicians should be aware of the diagnostic values of various symptoms, signs and antecedents. This information is particularly important in primary care settings, where sophisticated diagnostic approaches are not always feasible. The aim of the study is to determine the probability that various symptoms, signs, antecedents and tests predict urinary tract infection (UTI) in women.

**Methods:**

We conducted a systematic search of the MEDLINE and EMBASE databases to identify articles published in all languages through until December 2008. We particularly focused on studies that examined the diagnostic accuracy of at least one symptom, sign or patient antecedent related to the urinary tract. We included studies where urine culture, a gold standard, was preformed by primary care providers on female subjects aged at least 14 years. A meta-analysis of the likelihood ratio was performed to assess variables related to the urinary tract symptoms.

**Results:**

Of the 1, 212 articles identified, 11 met the selection criteria. Dysuria, urgency, nocturia, sexual activity and urgency with dysuria were weak predictors of urinary tract infection, whereas increases in vaginal discharge and suprapubic pain were weak predictors of the absence of infection. Nitrites or leukocytes in the dipstick test are the only findings that clearly favored a diagnosis of UTI.

**Conclusions:**

Clinical findings do not aid in the diagnosis of UTI among women who present with urinary symptoms. Vaginal discharge is a weak indicator of the absence of infection. The urine dipstick test was the most reliable tool for detecting UTI.

## Background

Clinicians should be aware of the diagnostic values of various symptoms, signs and antecedents. This information is particularly important in primary care settings, where sophisticated diagnostic approaches are not always feasible.

Urinary tract infection (UTI) is one of the most common bacterial infections seen in primary care, second only to infections of the respiratory tract [1,2]. Infections of the urinary tract can present with various symptoms and signs [3] and are particularly common among women, with an incidence of about 3-9% in young women [4,5] and 20% in women aged more than 65 years [6]. Approximately 2.692 of every 100, 000 American individuals were diagnosed with UTIs in the year 2000 [7]. In the United States, UTIs account for 2-3% of all visits to the general practitioner [8] (i.e., 7-8 million annual visits [1,9]) and 2% of all prescriptions [10]. This results in an annual cost of nearly 1.6 billion dollars [11,12]. Sixty-one percent of all UTIs are managed in the primary care setting [1,13].

There is an ongoing debate about the best way to diagnose UTIs in the primary health care setting [14]. This condition is often challenging to diagnose [15] because the clinician has to decide on the proper diagnostic tools and mode of interpretation [16] according to the diagnostic accuracy of clinical findings. Time and resources are scarce for primary health care professionals, whose services are in high demand. Thus, there is a need for studies of the effectiveness of diagnostic and therapeutic tools. A systematic review and meta-analysis of studies on the use of diagnostic tools in primary health care will help identify clinical findings that are useful in the diagnosis of UTIs.

Two meta-analysis [17,18] were performed to determine the usefulness of clinical findings in the diagnosis of UTI. Bent [17] analysis included data from a variety of settings such as hospitals, emergency departments, and specialty clinics. The findings revealed that the prevalence (i.e., *a priori *probability) of UTI differed depending of the clinical spectrum of patients with UTI. Giesen [18] estimated post-test values of some clinical findings used to diagnose UTI in primary care settings across three different threshold reference standards (10^2 ^or 10^3 ^or 10^5 ^CFU/ml). We reviewed the accuracy of various symptoms, signs, antecedents and tests performed in the primary care physician's office for the diagnosis of UTI.

## Methods

We conducted a systematic search of the MEDLINE (i.e., literature dating from 1966), and EMBASE (i.e., literature dating from 1974) databases for abstracts of articles published in all languages through December 2008. The selection of publications was made using the following six steps. First, we used an automated system to retrieve abstracts and references that contained the keywords indicated in Table 1. Second, we selected articles whose abstracts were consistent with a previously defined selection protocol. When data in the abstract were insufficient to determine if the article should be included in our study, or when the abstract was not available, the decision to include study was deferred to the next step. Third, we obtained full-text versions of the articles selected in the second step, including articles without abstracts or with insufficient data in the abstracts, and these publications were independently assessed by the two authors using a specific protocol to determine if they should be included in this review. At this step, a secondary review was performed using bibliography of each of the selected articles as a starting point, which included other studies. Fourth, we compared the decisions made by the reviewers during the third step, and discrepancies were discussed until a consensus was attained. Fifth, an external expert assessed those articles for which an agreement had not been reached. The two concordant decisions were those considered for inclusion or exclusion of the study. Sixth, we contacted the authors of articles with missing data or unclear findings to obtain further explanations. If the necessary data were not available, the article was excluded from our study. Table 2 describes the data of all included articles [14,19-28]. Our automated search was complemented by a manual search for papers that were not found in the databases, but that fulfilled our inclusion criteria. These papers were then retrieved using the references of the articles that had been found.

**Table 1:** Keywords used in the automated searches of Medline and EMBASE

Search 1	Parameters defining the type of study:
#1	diagnostic OR (diagnostic test) OR (clinical diagnostic) OR (medical diagnosis) OR (physical examinations) OR sensitivity OR specificity OR (likelihood ratio) OR prediction OR (predictive value) OR (reproducibility of results) OR (Bayes theorem)
**Search 2**	**Parameters defining the study's dependent variable:**
#2	(UTI) OR (urinary tract infection) OR (urinary infection) OR (urinary tract)

**Search 3**	**Parameters defining the predictive variables studied:**
#3	(urinary symptoms) OR (physical examination) OR signs OR (clinical history) OR dysuria OR (burning urination) OR (pain urination) OR (painful urination) OR frequency OR (urinary frequency) OR urgency OR (urinary urgency) OR (nocturnal incontinence) OR (diurnal incontinence) OR (burning sensation) OR (urinary tenesmus) OR (difficulty micturition) OR (vaginal discharge) OR (vaginal irritation) OR (genital discomfort) OR (perineal discomfort) OR (lower abdominal discomfort) OR (suprapubic tenderness) OR (hypogastric discomfort) OR (cold chills) OR (general malaise) OR dyspareunia OR (lumbar tenderness) OR (flank pain) OR (costovertebral angle tenderness) OR (lower abdominal pain) OR fever OR (genital erythema) OR (genital ulcers) OR (dipstick test) OR (dipstick urinalysis) OR pyuria OR (leukocyte esterase) OR nitrite OR hematuria OR (recurrent UTI) OR (history of UTI) OR (sexual complaints) OR (sexual activity) OR (diabetes mellitus) OR urolithiasis OR vulvovaginitis OR (STD) OR (sexually transmitted diseases) OR (urinary catheterization).

**Search 4**	**Parameters defining the setting of the study:**
#4	(primary care) OR (family practice) OR (general practice) OR (family medicine) OR (primary medicine) OR (primary health care).

**Search 5**	**Finally, the global scheme of the search therefore was including each of 4 searches mentioned above:**
	#1 AND #2 AND #3 AND #4

**Table 2:** Description of studies

Study/Year	Design	Participants	Tests and reference	Variables measured	Prevalence UTI
Verest LFHM ^(14)^ 2000	prospective	- 292 women suspect of UTI - age > 12 yr - primary care - excluded if antibiotic has been taken - Netherlands	-dipstick -culture	- nitrites and leukocytes in dipstick test (cut-off not defined) - culture (UTI if ≥10^5 ^CFU/mL)	p(UTI) 58% (168/292)

Baerheim A ^(19)^ 1999	Prospective symptoms were registered in the patient's home for three days	- 196 women with symptoms of the lower urinary tract - age 48 yr (average) - primary care - Norway	- self-monitoring of symptoms thrice daily for 3 days - dipstick test - culture	- dysuria - frequency - urge - suprapubic pain - low back pain - pyuria on dipstick - culture (UTI if ≥10^5 ^CFU/mL)	p(UTI) = 46.8%

Dawborn JK ^(20)^ 1973	prospective	- 62 women with symptoms suggestive of UTI - age 15-50 yr - primary care - Victoria (Australia)	- questionnaire for the register of antecedents, symptoms and signs - culture	- history of urinary complaints - pain on micturition - frequency - back pain - anorexia - vaginal irritation - abundant vaginal discharge - culture (UTI if ≥10^5 ^CFU/mL)	p(UTI) = 63% (37/59)

Fahey T ^(21)^ 2003	Prospective	- 136 women with symptoms for UTI - age not indicated - primary care - United Kingdom	- asked about explicity for 11 symptoms associated with UTI - near patient test - culture	- frquency and dysuria - dysuria - fequency - haematuria - urgeny - nocturia - nauses - vomiting - abdominal pain - back pain - vaginal discharge - leukocyte near patient test - nitrite - culture (UTI if ≥10^5 ^CFU/mL)	p(UTI) = 38%

Fairley KF ^(22)^ 1971	prospective	- 78 women with symptomatic micturition - age 8-80 yr, most 30-40 yr - primary care - Victoria (Australia)	- symptoms and signs were registered systematically - urine culture - bladder catheterized depending on culture results	- frequency - burning - suprapubic pain - loin pain - temperature - rigors - nausea and vomiting - haematuria (microscopy) - culture (UTI if ≥10^4 ^bacteria/mL)	p(UTI) = 71% (55/78)

Jellheden B ^(23)^ 1996	systematic register of clinical findings from all patients	- 819 women with signs and symptoms suggesting UTI - age over 18 yr - primary care - Sweden	- dipstick test - urinoculture	-dysuria -frequency -suprapubic pain -flank pain -fever -nitrites on dipstick - culture (agar, UTI if ≥10^5 ^CFU/mL)	p(UTI) = 83%

Lawson DH ^(24)^ 1973	prospective	-343 women with symptomatic micturition - age 15-55 yr - Canada	- use of a questionnaire to register data - urine sample - urinoculture - control of evolution after 14 days	- pyrexia - loin pain - frequency - dysuria - nocturia - stress incontinence - previous symptoms - culture (UTI if ≥10^5 ^CFU/mL)	p(UTI) = 34.3%

Leibovici L ^(25)^ 1989	prospective	- 266 women with dysuria - 17 to 25 years old - primary care - Israel	- clinical interview and examination followed a check-list - urine sample taken for dipstick and culture	- sexual activity - vaginal discharge - duration of symptoms - pyuria (sediment) - hematuria (sediment) - culture (agar, UTI if ≥10^5 ^CFU/mL)	p(UTI) = 55.3%

Medina-Bombardó D ^(26)^ 2003	prospective	- 343 women with incident urinary symptoms - Median age 44 years old, (range 15-90) - Primary care - Spain	- clinical systematically interview and clinical exam by check-list - urine reactive strip test - urinoculture	- urinary symptoms: freqúency, burning, tenesmus, urgency, painfou voiding, difficult, diurnal and or nocturnal incontinence and combinations two of them. - medical history: urinary tract infections, sexual activity, vulvovaginitys, urolithiasis, diabetes mellitus, urinary catheterization - sings: lower abdominal pain, positive fist percussion, genital erythema, fever - accompanying symptoms: lower abdominal discomfort, general malaise, genital discomfort, dyspareunia, cold chills, uncreased vaginal discharge, perineal discomfort. - reactive strip test: pyuria, nitrite - culture (agar, UTI if ≥10^5 ^CFU/mL)	p(UTI) = 48.4%

Nazareth I ^(27)^ 1993	prospective	- 61 women presenting with symptoms on urination (frequency or dysuria) - 16-45 years old - primary care - no antibiotic treatment - United Kingdom	- data on symptoms, signs and clinical exam were collected by the physician using a questionnaire with open questions - urine sample for culture	- dysuria - frequency - urgency - vaginal symptoms - abdominal symptoms - back pain - haematuria - fever - culture (≥10^5 ^CFU/L)	p(UTI) = 28%

Osterberg E ^(28)^ 1996	prospective	- 217 women with dysuria and/or urgency/frequency - age 15-35 yr - primary care - Sweden	- questionnaire on symptoms - dipstick - culture on "Dipslide"	- dysuria and urgency/frequency - nitrites on dipstick - positive granulocyte esterase (≥1) - culture (UTI if ≥10^5 ^CFU/mL)	p(UTI) = 52%

UTI urinary tract infection; CFU/mL Colony Forming Units/milliliter sample; p(UTI) prevalence of UTI

### Inclusion criteria

All articles included in the review provided implicit or explicit evidence obtained from women aged 14 years and older who presented to their primary health care physician with urinary tract complaints of recent onset that had not yet been treated. We included original, observational, prospective, diagnostic studies of the accuracy of clinical findings (i.e., symptoms, signs and antecedents) with regards to the diagnosis of UTI. The studies included consecutive cases of primary care patients in whom UTI was suspected. Review articles and meta-analyses were considered only as sources of references for the original studies. All clinical findings were collected systematically, according to defined standards (i.e., check-list protocol or similar). The variables assessed in the studies were consistent with clinical parameters related to UTI. The cut-off values for urine cultures, our gold standard, were provided (in CFU/mL) for the infecting pathogen, based on usual agar plate urine culture. Data needed to calculate the sensitivity, specificity, and/or likelihood ratio for the predictive value of symptoms, signs, antecedents and/or results of the urine dipstick test was available for all studies included in this report. Finally, the studies included a sufficient period of follow-up to assure that patients' illness is attributable to UTI.

### Exclusion criteria

Studies were excluded from our analysis if the study population included fewer than 50 subjects; a poorly defined population that is, when context, gender, age or study subjects inclusion criteria were not specified in the methods section. When population was recruited from a hospital or a specialty practice, patients younger than 14 years, or patients whose urinary symptoms were not of recent onset (more than one month since onset of symptoms). Studies were also excluded for non-systematic assessment of clinical findings that is, when authors did not describe specifications of materials and methods involved, including how and when measurements were taken. Also were excluded those studies with non-consecutive or non-randomized recruitment of patients into the study, or unoriginal data.

After studies selection we identified that most of studies used as cut off > 10^5 ^CFU/mL. In order to avoid heterogeneity of the study population and disease definition as well as statistical power both authors agreed to exclude bacterial count cut off < 10^5 ^CFU/mL.

### Quality assessment

The QUADAS assessment tool [29] was applied by one of the reviewers (DMB) in order to evaluate the quality of included studies.

### Analysis

The likelihood ratio (LR) is the ratio of two probabilities, namely the probability that a specific test result is obtained in patients with the disease divided by the probability of a test result in patients without the disease. Positive likelihood ratios (PLRs) are calculated for positive test results and negative likelihood ratios (NLRs) are calculated for negative test results. The diagnostic odds ratio (DOR) describes how well the test works in subjects with disease compared with subjects without disease, as well as the discriminatory properties of positive and negative test results (PLRs and NLRs, respectively). Every clinical exam finding was considered a different test to aid in the diagnosis of urinary tract infections. Sensitivity (S), specificity (Sp), positive and negative likelihood ratios (PLR and NLR) and (DOR), as well as their corresponding standard errors and 95% confidence intervals (CI_95%_), were calculated for every symptom or sign. Results were analyzed for all possible synonyms of each variable, as well as the more commonly used synonyms. Summary or pooled LR (PLRp, NLRp) and pooled DOR (DORp) indices, as well as their corresponding standard errors and CI_95%_, were calculated when two or more studies described the same clinical finding. The statistical heterogeneity of the LR and DOR indices was analyzed. When heterogeneity was found, the threshold effect was analyzed using the Moses-Shapiro-Littenberg method. If heterogeneity could not be explained by the threshold effect, the data were analyzed using a meta-regression model that included prevalence as an independent variable. When the summary likelihood ratio was estimated (i.e., when clinical data were obtained from different studies), heterogeneity was assessed via Chi-square tests and I^2 ^inconsistency tests. Indices were pooled for the fixed effects method, when possible, and heterogeneity was rejected. If heterogeneity or inconsistency could not be explained, the pooled likelihood ratio was interpreted cautiously. The summary LR for random effects is usually recommended for the analysis of accuracy studies when an estimate of between study variation can be incorporated. All statistics were calculated using the Meta-DiSc [30] and RevMan 4.2 [31] software.

## Results

A total of 1, 212 articles were retrieved via an automated search (1, 059 from the MEDLINE database, 242 from the EMBASE database, 89 found in both databases). We excluded studies of urinary tract treatments, reviews, diagnostic studies performed in pediatric settings, studies including men, editorials, and articles addressing issues other than UTI. Seventy-eight articles were preselected during the initial screening of abstracts.

**Figure 1:**
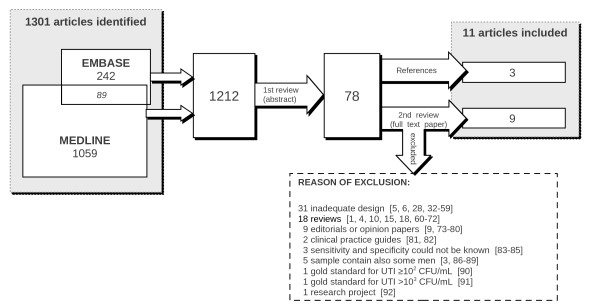
**Summary of exclusion of studies**.

The second screening (of full-text articles) revealed eight studies [14,18-23,26,28] that satisfied the inclusion criteria. Three additional articles were cited in reviews or in the references of other articles and were subsequently included in our study [24,25,27] (Figure 1). Reasons for exclusion are shown in Figure 1[1,3-6,9,10,15,18,28,32-92]. We contacted eight authors to obtain missing data [2,21,23,26,83,85,86,90], but only four authors replied [21,23,26,85].

The quality assessment of included studies is presented in Table 3. The index test was not described in sufficient detail to permit replication in all studies included and the reference standard in seven. Blinding of both index test results and reference test was poorly reported in the 10 of the 11 studies considered. In all studies patients received the same reference standard.

**Table 3:** Quality analysis by QUADAS tool of all studies included

	Reference number of studies analysed											
	**QUADAS item**	[14]	[19]	[20]	[21]	[22]	[23]	[24]	[25]	[26]	[27]	[28]

1.	Was the spectrum of patients representative of the patients who will receive the test in practice?	Y	Y	Y	Y	U	Y	Y	Y	Y	Y	Y

2	Were selection criteria clearly described?	U	Y	N	N	U	U	N	Y	Y	Y	Y

3.	Is the reference standard likely to correctly classify the target condition?	Y	Y	Y	Y	Y	Y	Y	Y	Y	Y	Y

4.	Is the time period between reference standard and index test short enough to be reasonably sure that the target condition did not change between the two tests?	U	Y	U	U	U	U	Y	U	Y	Y	Y

5.	Did the whole sample or a random selection of the sample, receive verification using a reference standard of diagnosis?	Y	Y	U	Y	Y	Y	Y	Y	Y	Y	Y

6.	Did patients receive the same reference standard regardless of the index test result?	Y	Y	Y	Y	Y	Y	Y	Y	Y	Y	Y

7.	Was the reference standard independent of the index test (i.e. the index test did not form part of the reference standard)?	Y	Y	U	U	U	U	U	Y	Y	U	Y

8.	Was the execution of the index test described in sufficient detail to permit replication of the test?	N	N	N	N	N	N	N	N	N	N	N

9.	Was the execution of the reference standard described in sufficient detail to permit its replication?	N	N	Y	N	N	Y	N	Y	N	N	Y

10.	Were the index test results interpreted without knowledge of the results of the reference standard?	U	U	U	U	U	U	U	U	Y	U	U

11.	Were the reference standard results interpreted without knowledge of the results of the index test?	U	U	U	U	U	U	U	U	Y	U	U

12.	Were the same clinical data available when test results were interpreted as would be available when the test is used in practice?	Y	Y	Y	Y	Y	Y	Y	Y	Y	Y	Y

13.	Were uninterpretable/intermediate test results reported?	N	N	N	N	N	N	N	N	N	N	N

14.	Were withdrawals from the study explained?	N	Y	N	Y	N	N	N	N	Y	Y	N

Y = item fulfilled N = item not fulfiled U = item unclearly fulfilled

**Figure 2:**
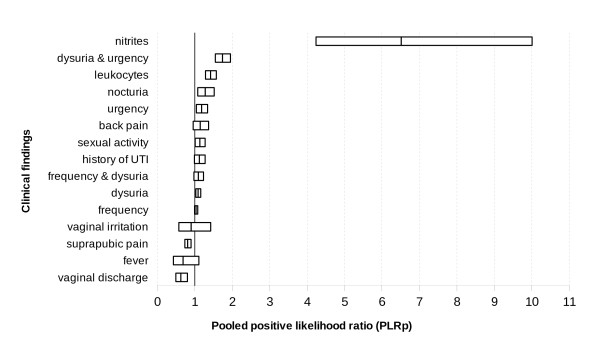
**Comparative estimate of the number of times a woman with a urinary tract infection (UTI) is more likely than a woman without a UTI to present with certain clinical findings (i.e., pooled positive likelihood ratios - PLRp - and confidence intervals for all clinical findings)**.

**Figure 3:**
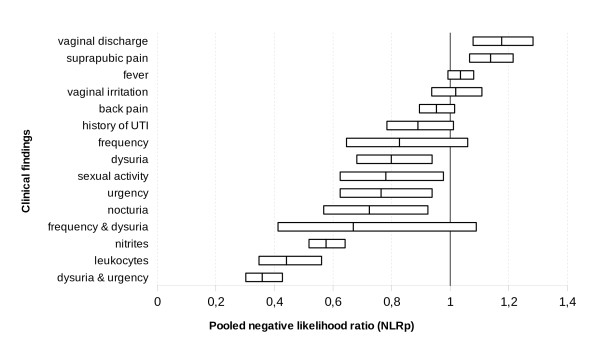
**Comparative estimate of the number of times a woman with a urinary tract infection (UTI) is more likely than a woman without a UTI to lack certain clinical findings (i.e., pooled negative likelihood ratios - NLRp - and confidence intervals for all clinical findings)**.

**Figure 4:**
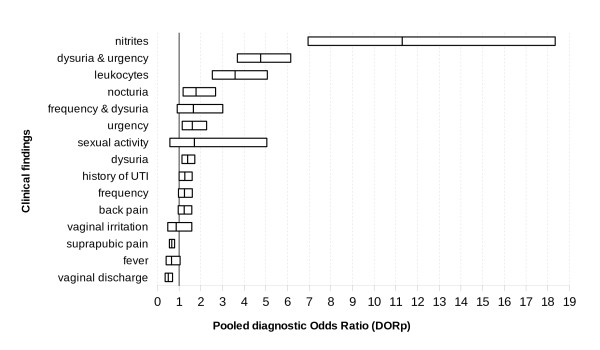
**Comparative usefulness of clinical findings in the diagnosis of urinary tract infections**. The pooled diagnostic odds ratio (DORp) values estimate the accuracy of the test (i.e., clinical finding) in patients with and without disease, as well as the discriminatory properties of positive and negative test results (PLR and NLR, respectively).

We performed a meta-analysis of the studies shown in Table 2 to determine the likelihood ratios (LR) for symptoms such as dysuria, urination frequency, urinary urgency, nocturia, back pain, suprapubic pain, fever, increased vaginal discharge, vaginal irritation, history of UTI, sexual activity and the presence of nitrites or leukocytes in the urine dipstick test, see Table 4. Figures 2, 3 and 4 show the comparative PLRp, NLRp and DORp, respectively, for all clinical findings.

**Table 4:** Pooled positive and negative likelihood ratio and diagnostic odds ratio whith theirs 95% conficence interval, inconsitency index and heterogeneity chi-squared significance for all clinical findings analized

Clinical findings analized	Number of studies	Sample size	Pooled Positive Likelihood Ratio			Pooled Negative Likelihood Ratio			Pooled Diagnostic Odds Ratio		
			**PLRp (95% CI)**	**% I^2^**	**p (Chi^2^)**	**NLRp (95% CI)**	**% I^2^**	**p (Chi^2^)**	**DORp (95% CI)**	**% I^2^**	**p (Chi^2^)**

Dysuria	8	1862	1.09 (1.03 - 0.16)	41.7	0, 1	0.80 (0.68 - 0.94)	0	0, 613	1, 40 (1, 13-1, 73)	0	0, 470

Frequency	8	1861	1.03 (0.99 - 1.08)	63.8	0, 007	0.83 (0.65 - 1.06)	49.3	0, 055	1, 25 (0, 97-1, 61)	52, 2	0, 041

Suprapubic pain	7	2409	0.81 (0.73 - 0.89)	63.4	0, 012	1.14 (1.07-1.21)	40.8	0, 119	0, 66 (0, 56 - 0, 79)	49, 7	0, 064

Back pain	7	1512	1.15 (0.96-1.37)	26.5	0, 227	0.95 (0.90 - 1.01)	0	0, 6	1, 24 (0, 96 - 1, 59	15, 9	0, 309

History of UTI	4	998	1.23 (0.99 - 1.27)	59.3	0, 061	0.89 (0.79 - 1.01)	41.6	0, 162	1, 27 (1, 00 - 1, 60)	52, 1	0, 1

Fever	4	797	0.69 (0.43 - 1.11)	36.1	0, 195	1.04 (0.99 - 1.08)	51.9	0, 101	0, 65 (0, 41 - 1, 05)	39, 0	0, 178

Vaginal discharge	4	722	0.63 (0.49 - 0.80)	0	0, 428	1.18 (1.08 - 1.28)	78.7	0, 003	0, 50 (0, 36 - 0, 70)	0	0, 401

Leukocytes *	4	705	1.42 (1.23 - 1.57)	87.8	< 0, 001	0.44 (0.35 - 0.56)	0	0, 516	3, 58 (2, 53 - 5, 07)	0	0, 914

Urgency	4	577	1.18 (1.04 - 1.34)	28.3	0, 242	0.75 (0.62 - 0.94)	89.3	< 0, 001	1, 61 (1, 15 - 2, 27)	40, 5	0, 163

Nitrites *	3	626	6.51 (4.24 - 10.01)	65.2	0, 056	0.58 (0.52 - 0.64)	58.0	0, 093	11, 3 (6, 95-18, 35)	51, 6	0, 126

Dysuria & urgency ^§^	2	1149	1.53 (0, 94 - 2.50)	94.2	< 0, 001	0.44 (0.21 - 0.92)	89.9	0, 002	3, 47 (1, 04 - 11, 62)	92, 0	< 0, 001

Sexual activity ^§^	2	584	1.14 (0, 90 - 1.44)	75.0	0, 05	0.66 (0.28 - 1, 58)	89.8	0, 002	1, 71 (0, 58 - 5, 05)	87, 6	0, 004

Nocturia	2	415	1.28 (1.08 - 1.52)	0	0, 955	0.72 (0.57 - 0.92)	0	0, 508	1, 79 (1, 17 - 2, 69)	0	0, 777

Vaginal irritation	2	361	0.90 (0.57 - 1.42)	36.3	0, 210	1.02 (0.94 - 1.11)	0	0, 373	0, 87 (0, 48 - 1, 59)	17, 6	0, 271

Frequency & dysuria	2	284	1.10 (0.98 - 1.23)	39.3	0, 199	0.67 (0.41 - 1.09)	77.2	0, 036	1, 66 (0, 91 - 3, 02)	72, 7	0, 056

PLRp (95% CI) Pooled Positive Likelihood Ratio and its 95% confidence interval

NLRp (95% CI) Pooled Negative Likelihood Ratio and its 95% confidence interval

DORp (95% CI) Pooled Diagnostic Odds Ratio and its 95% confidence interval

I^2 ^Inconsitency Index

p (Chi^2^) heterogeneity chi-squared significance

* Measured by Dipstick test in urine sample

^§ ^Index calculated by random effects model (else by fixed effects model)

Dysuria, urgency, nocturia, sexual activity and the simultaneous presence of urgency and dysuria were weak diagnostic indicators of UTI. However, an increase in vaginal discharge and suprapubic pain were weak predictors of the absence of infection. Frequency of urination, back pain, fever, vaginal irritation, history of UTI, as well as the simultaneous presence of dysuria with urgency were not significant indicators of UTI. Nitrites or leukocytes in the dipstick test were the only findings that clearly indicated the presence of UTI.

The studies included in our meta-analysis were statistically homogeneous, with regards to the PLRp of all variables except frequency of urination, suprapubic pain, sexual activity and the presence of leukocytes in the urine, as indicated by the dipstick test. NLRp was homogeneous for nine variables (i.e., dysuria, frequency, nocturia, back or suprapubic pain, fever, history of UTI, vaginal irritation and the presence of nitrites in the urine, as indicated by the dipstick test).

The threshold effect can explain LR heterogeneity indexes with regards to urgency, frequency of urination, suprapubic pain, and the presence of leukocytes in the urine, as indicated by the dipstick test. We were unable to analyze threshold effect of data on sexual activity, vaginal irritation, and the simultaneous presence of dysuria with urgency or frequency because only two studies assessed both of these variables. Prevalence was not a source of heterogeneity in the metaregression model.

Only two studies studied nocturia (415 cases), vaginal irritation (361 cases), dysuria with frequency (284 cases) or urgency (1, 149 cases), or sexual intercourse (584), consequently, it was not possible to explore their threshold effect by Moses-Shapiro-Littenberg method. There is also considerable inconsistency among studies with regards to the PLRp and NLRp values for sexual activity (I^2 ^= 75.0% PLRp and 89.8% NLRp) and for the simultaneous presence of dysuria and urgency (94.2% PLRp and 89.9% NLRp). However, there were inconsistencies in the analysis of PLRp in the cases presence of leukocyes (87.8%), frequency (63.8%) and suprapubic pain (63.4%), as well as in the analysis of NLRp in the cases of urgency (92.8%), vaginal discharge (78.7%) or the simultaneous presence of dysuria and frequency (77.2%).

## Discussion

Our results show that exploratory clinical findings may suggest a diagnosis of UTI in women; however, nitrituria is clearly the most useful diagnostic indicator. Some clinical findings, alone or in combination with others, can indicate the presence of UTI. Consistent with the results of a meta-analysis by Bent [17], our results show that dysuria and the presence of nitrites or leukocytes in the urine, as indicated by the dipstick test, are useful in the diagnosis of UTI. Furthermore, increased vaginal discharge indicates the absence of infection. The presence or absence of back pain provides little diagnostic information. However, contrary to our results, Bent [17] found that frequent urination or back pain increased the likelihood of UTI, whereas vaginal irritation was not associated with UTI. Urgency, nocturia, sexual activity and a history of UTI were not considered by Bent, whereas costovertebral angle tenderness was not considered in our study. Contrary to our analysis, Bent calculated separate pooled indexes for back pain and flank pain. Both metaanalyses share several of the above mentioned shortcomings; thus, these results should be interpreted with caution. Bent's meta-analysis [17] included a considerably more heterogeneous population than did our study, but did not include any analysis of heterogeneity. Bent's pooled LR indexes were calculated using a random effects model, whereas ours were calculated using a fixed effects model with corresponding differences in confidence intervals.

On the other hand in Giesen meta-analysis [18] only dysuria and urgency pooled LR were favorable at UTI diagnostic and fever were indifferent as observed in our results, using the same threshold in urinoculture. Other results were not in accordance with our finding. That is, lower abdominal pain PLR and NLR were indiferent in Giesen meta-analisis and PLR favourable and NRL indiferent for us. Giesen's pooled LR indexes conficence intervals were calculated using random effects model and we used fixed effects.

We would like to emphasize the fact that we analyzed only those variables for which results were found in more than one study. The studies included in our analysis were of adequate quality with regards to all concepts usually assessed [17,93] with the exception of one study that included a sample size of 48 subjects. However, that study met all other quality requirements [20]. Although we may have lost some relevant clinical information by excluding studies with sample sizes less than 50, we gained statistical precision and our data demonstrated minor variability when the random effects method was applied. In addition, our quality analysis revealed a decrease in heterogeneity.

We included studies that recruited consecutive samples, which is the recommended method [93] to reflect clinical conditions. Randomized samples would be ideal; however, there have not been published studies of diagnostic tests based on randomized samples.

Variability in the results of a diagnostic test can be attributed to several factors. The sensitivity and specificity of a test, as well as the LR, may change during the course of the disease [93-95] as its manifestations become more or less pronounced. Validity test parameters such as sensitivity, specificity and likelihood ratio remain constant only if the test is used on a population with similar characteristics as the subjects whose parameters were originally estimated. However, post-test probability depends on pre-test probability. This may be related to the variability encountered when the same diagnostic tool is used in different levels of health care, a phenomenon known as the referral filter bias [96,97]. This concept is independent of the variability that results from differences in prevalence at one stage of the natural history of the disease [93,94,96,97], and it may also affect the predictive value of the test when used in that context. Therefore, we only included studies performed in the primary care setting. This may partially explain the differences between our results and those reported by Bent [17], whose population was more heterogeneous.

Another source of variability is interobserver variability, due to differences in the understanding of terms and in the examination techniques used by different field researchers. In many instances, variables are not clearly defined (i.e., different names may refer to the same concept, whereas one name may refer to quite different things), making it difficult to understand what the study is attempting to measure. Interobserver variability is a source of potential bias and an important limitation to studies that aim to estimate the validity of diagnostic tools. It is also of particular concern in studies that focus on symptoms. There were no clear definitions of variables in the studies included in our analysis. For example, pain in the lumbar region was variously referred to as *back pain *[20,21,27], *low back pain *[18], *loin pain *[22,87,88], *flank pain *[23] or *kidney/flank pain *[90] while other authors [17] have distinguished *flank pain *from *back pain*. Fever was referred to as *fever *[26,27,87,88], *pyrexia *[24] or *temperature *[22]. However, for the poorly defined symptom dysuria, the overall PLRp and NLRp values obtained for *dysuria *[18,21,23,24,27] were 1.06 (CI_95% _1.00-1.13) and 0.84 (CI_95% _0.68-1.03), respectively. These findings do not differ greatly from the results obtained when we include some of the terms usually considered synonymous for dysuric syndrome [98,99], such as *pain on micturition *[20,26] or *burning on micturition *[22]. The PLRp and NLRp values would then be 1.09 (CI_95% _1.03-1.16) and 0.80 (CI_95% _0.68-0.94), respectively.

The evidence power analysis further revealed homogeneous DORp values for urgency, vaginal discharge, leukocytes in the urine, and simultaneous dysuria and frequency. However, the DORp values were heterogeneous for frequency, sexual activity and simultaneous dysuria and urgency. In all cases, the threshold effect could be explained by heterogeneity, except for history of UTI. We included studies that showed wide differences in the pre-test probability of UTI (i.e., range: 28% [27] to 83% [23]) because the target populations in all of these studies were similar (i.e., primary care patients). An unknown factor might explain these differences, but pre-test probability could not account for the heterogeneity observed in a metaregression model. The available data do not allow us to classify our results according to age for the comparison of the pre-test probabilities of UTI [7,100-102]. Poor descriptions of methods and incomplete data made it difficult to assess the methodological quality of the studies included in our analysis, to compare our inclusion and exclusion criteria, and to identify and control for other characteristics that may not have been reflected in the publication. The validity of our meta-analysis is limited by quality analysis of selected studies. Our most important limitations are the poor definition of the index test, which is clinical symptoms and signs, in all studies. Furthermore, unclear specification of blind interpretation of the index test or reference standard results. Consensus tools like QUADAS [29,103] are needed to assess the quality of studies included in meta-analysis such as this and necessary to performing diagnostic test studies designs. Therefore, it is difficult to exclude the possibility of work-up bias, diagnostic-review bias, test-review bias or incorporation bias [104], all of which could have influenced the selection or classification bias of the studies included in our analysis. Moreover, these problems limit our capacity to detect a bias that is particularly difficult to control in systematic reviews of diagnostic tests; namely, publication bias [105,106]. However, the existence of publication bias can be suspected. An interesting project [92] promises to assess the validity of clinical findings and improve their use in diagnosing UTI.

## Conclusions

### Implications for practice

Clinical findings are not '*per se*' predictive of UTI. In women who present to their primary care provider with urinary symptoms, an office urine dipstick test could be helpful to guide UTI diagnosis, and identification of nitrites or leukocytes is a good predictor of UTI.

### Implications for research

The quality of meta-analyses and systematic reviews of diagnostic tests could be improved by the development of consensus tools. These tools would aid in the design of better studies and in the analysis of clinical findings, which would ultimately improve the diagnosis of UTI.

## Competing interests

The authors declare that they have no competing interests.

## Authors' contributions

DMB participated in the coordination and design of the study, in search, review and selection of articles, perfomed the statistical analysis and drafted the manuscript. AJP participated in the design of the study, in review an selection of articles and helped to draft the manuscript. Both authors read and approved the final manuscript.

## Pre-publication history

The pre-publication history for this paper can be accessed here:

http://www.biomedcentral.com/1471-2296/12/111/prepub
